# Macrophagic myofasciitis and subcutaneous pseudolymphoma caused by aluminium adjuvants

**DOI:** 10.1038/s41598-020-68849-8

**Published:** 2020-07-16

**Authors:** Hyunhee Kim, Ka Young Lim, Jeongwan Kang, Jin Woo Park, Sung-Hye Park

**Affiliations:** 1 Department of Pathology, Seoul National University Hospital, Seoul, 03080 Republic of Korea; 2 Department of Pathology, Seoul National University College of Medicine, Seoul, 03080 Republic of Korea; 3 Institute of Neuroscience, Seoul National University College of Medicine, Seoul, 03080 Republic of Korea; 4 Department of Pathology, Seoul National University College of Medicine, Seoul National University Hospital, 103 Daehak-ro, Jongno-gu, Seoul, 110-799 Republic of Korea

**Keywords:** Diseases, Medical research, Neurology, Pathogenesis, Signs and symptoms

## Abstract

Aluminium hydroxide is a well-known adjuvant used in vaccines. Although it can enhance an adaptive immune response to a co-administered antigen, it causes adverse effects, including macrophagic myofasciitis (MMF), subcutaneous pseudolymphoma, and drug hypersensitivity. The object of this study is to demonstrate pediatric cases of aluminium hydroxide-induced diseases focusing on its rarity, under-recognition, and distinctive pathology. Seven child patients with biopsy-proven MMF were retrieved from the Seoul National University Hospital (SNUH) pathology archives from 2015 to 2019. The medical records and immunisation history were reviewed, and a full pathological muscle examination was carried out. The mean age was 1.7 years (8.9–40 months), who had records of vaccination against hepatitis B, hepatitis A, and tetanus toxoid on the quadriceps muscle. The chief complaints were muscle weakness (n = 6), delayed motor milestones (n = 6), instability, dysarthria, and involuntary movement (n = 1), swallowing difficulty (n = 1), high myopia (n = 1), and palpable subcutaneous nodules with skin papules (n = 1). Muscle biopsy showed MMF (n = 6) and pseudolymphoma (n = 1) with pathognomic basophilic large macrophage infiltration, which had distinctive spiculated inclusions on electron microscopy. The intracytoplasmic aluminium was positive for PAS and Morin stains. Distinctive pathology and ultrastructure suggested an association with aluminium hydroxide-containing vaccines. To avoid misdiagnosis and mistreatment, we must further investigate this uncommon condition, and pharmaceutical companies should attempt to formulate better adjuvants that do not cause such adverse effects.

## Introduction

Despite the availability of several vaccine adjuvants, these based on aluminium hydroxide (a crystalline compound) continues to be widely used worldwide^1^. To date, the benefit of aluminium hydroxide-based adjuvants is not fully understood, in spite of its long-term usage. It has an enhancing effect on the adaptive immune response to a co-administered antigen through repository and pro-phagocytic effects and proinflammatory NOD-like receptor protein 3 (NLRP3) pathway activation^1^. NLR proteins are involved in the immune response and help in activating and regulating responses to injury, toxins, or invasion by microorganisms.

However, aluminium hydroxide has adverse effects, including macrophagic myofasciitis (MMF) and adverse drug reactions (ADRs) to aluminous adjuvants, which include aluminium neurotoxicity, autism spectrum disorder, and autoinflammatory syndrome^1,2^. Currently, tetanus, hepatitis A, hepatitis B, human papillomavirus, Haemophilus influenzae type B, pneumococcal and meningococcal vaccines, and anthrax vaccines use aluminium hydroxide as an adjuvant^3^.

MMF is an unusual inflammatory myopathy found in patients with arthromyalgia and muscle weakness that develops several months to years after administering aluminium-containing vaccines. However, symptoms immediately developed after vaccination have also been reported^4, 5^. Since Gherardi et al. first described MMF in 1998, most reported patients have been adult, although several articles have documented child cases^2,4–8^. The persistence of aluminium-based vaccine adjuvants significantly contributes to MMF pathogenesis^9^. Pathologically, massive infiltration by aluminium-containing macrophages is the diagnostic hallmark of MMF. These macrophages have obvious and distinctive features, i.e., a large cytoplasm filled with granules containing aluminium hydroxide, which are weakly basophilic on hematoxylin and eosin (H&E) staining, and Periodic Acid Schiff (PAS)-positive. Lymphocyte infiltration is also present in the perimysium or the perivascular area^1,10^. Other aluminium adjuvant-associated diseases include chronic fatigue syndrome^11^, autoimmune (autoinflammatory) syndrome induced by adjuvants (ASIA)^12,13^, and Gulf War syndrome (GWS) developed in soldiers following multiple aluminium-containing vaccinations^14,15^. Therefore, MMF itself is aluminium hydroxide induced granulomas in the vaccine injected sites, but it is not a local lesion but manifests severe systemic disease.

This study aimed to delineate the pathology of aluminium hydroxide-induced MMF and pseudolymphoma. We evaluated 7 cases of aluminium hydroxide containing vaccination-induced disease (six MMF and one pseudolymphoma) from the Seoul National University Hospital (SNUH) pathology archives from 2015 to 2019. The institutional review board (IRB) of our hospital approved this study (1812-070-994), and the informed consent from the parents was waived from our hospital IRB by retrospective review of the anonymized medical records, including pathology slides under the Korean Bioethics and safety act. All experiments were performed in accordance with Helsinki and human research protection program guidelines and regulations.

## Materials and methods

Seven child patients with biopsy-proven MMF were retrieved from the Seoul National University Hospital (SNUH) pathology archives from 2015 to 2019. The authors reviewed the medical records and data from H&E staining, immunohistochemistry slides, and electron microscopy examinations for each patient. Emphasis was placed on muscle pathology and the history of immunisation with aluminium-containing vaccines, namely hepatitis B virus (HBV), hepatitis A virus (HAV), and tetanus toxoid (TT) vaccines, which are usually administered to most new-borns in the Republic of Korea.

Six patients underwent muscle biopsy, while one patient was performed the skin and subcutaneous tissue biopsy. Half of the skeletal muscle tissue from all 6 patients was frozen for enzyme histochemistry. Several small pieces of the remaining tissue were fixed in 2.5% glutaraldehyde for electron microscopy, and the rest was fixed in 10% neutral formalin for paraffin embedding and H&E staining. Additionally, enzyme histochemistry, NADH-TR, modified Gomori, Periodic Acid–Schiff (PAS), ATPase (pH 4.3, 4.6, and 9.4), and succinic dehydrogenase tests were conducted. Immunohistochemical staining against CD68, CD56, CD3, CD20, CD4, and CD8 was performed on formalin-fixed and paraffin-embedded (FFPE) sections in all cases. Morin stain was conducted on FFPE sections with Morin hydrate (MACOB-5G, Sigma Aldrich-Merck) and Sudan black B (199664-25G, Sigma Aldrich-Merck), as well as DAPI, which was examined under immunofluorescence microscope according to the previous report^16^. Supplementary Table 1 lists the sources of the primary antibodies used. Immunohistochemical stains were carried out with the BenchMark ULTRA system (Roche Diagnostics). For antibody control, we used proper positive control tissue and omitted the primary antibody for negative control. Electron microscopic study was performed in all 7 cases and processed routinely as described in the previous paper. The examination was done with a Jeol transmission electron microscope (JEM1400).

### Ethical approval

The institutional review board of our hospital (SNUH) approved this study (1812-070-994).

## Results

The mean patient age at the time of diagnosis by muscle or tissue biopsy was 1.7 years (8.9–40 months). The male to female ratio was 1:1.3. The chief complaints were delayed motor milestones (n = 6), muscle weakness (n = 6), including congenital hypotonia (n = 1). Sensorineural hearing loss (n = 1), involuntary movement, instability, and frequent falling (n = 1), and tracheo-broncho-laryngomalacia (n = 1) were present in each patient. One patient with subcutaneous tissue involvement only did not have subjective symptoms. In case 2, who presented with delayed motor milestones and muscle weakness, brain MRI showed germinal matrix and intraventricular haemorrhage, suggesting the clinical manifestation of Fukuyama muscular dystrophy, although no *FKTN* gene alteration was detected in a gene panel study tailored to childhood muscle diseases. In case 4, presenting with an involuntary movement, instability, and frequent falling, brain MRI at the end of 21 months of age revealed a T2 high-signal intensity lesion on the bilateral basal ganglia. CK elevation (2,743 IU/L) was present in only one MMF patient (case 2). History of fever was mentioned in the hospital record of case 5, but ESR or CRP elevation was absent in all patients. Table 1 lists the summary of the clinicopathological data from these seven patients.

**Table 1:** Summary of the clinical and pathological findings of our series of MMF.

Case	Age at muscle biopsy (months/gender)	Symptoms for muscle biopsy	Accompanying clinical manifestations	Clinical impression	CK (IU/L, Normal range 20-270 IU/L)	Pathological diagnosis	Spiculated inclusions on EM	Vaccination history	Delay from vaccine (months)	Current age (mo) and status
1	21/F	Congenital hypotonia, muscle weakness, brace	Delayed motor milestone, swallowing difficulty	R/O Mitochondrial disorder	71	MMF with some T- & B-cell infiltration	Present	DPT Hepatitis B	8	64.3/unable to walk, but possible to sit with aid
2	8.9/M	Muscle weakness,	Delayed motor milestone, ventricular and germinal matrix hemorrhage, High myopia	R/O congenital myopathy, Known POMGNT1 mutation related alpha dystroglycan defect* No FKTN mutation **	2,743	MMF with some T- & B-cell infiltration	Present	DPT Hepatitis B	3	51.8/unable to walk
3	10.7/M	Muscle weakness,	Delayed motor milestone, sensorineural hearing loss, unable to sit or work	R/O Mitochondrial disorders	122	MMF	Present	DPT Hepatitis B	5	46.9/ unable to sit or walk, severe constipation
4	29.8/F	Muscle weakness	Delayed motor milestone, Instability, dysarthria, involuntary movement	R/O Mitochondrial disorders, autoimmune encephalitis	67	MMF with some T- & B-cell infiltration	Present	DPT Hepatitis B	23	56.2/instability, severe dysarthria, unable to stand or walk
5	15.7/M	Muscle weakness,	Delayed motor development, Bronchio-tracheo-laryngomalacia	R/O Congenital myopathy	58	MMF	Present	DPT Hepatitis B	9	27.2/able to walk with walking aid, muscle weakness
6	21.6/F	Muscle weakness of low extremities	Delayed motor milestone, frequent fall down	R/O Spinal muscular atrophy, congenital myopathy, congenital muscular dystrophy	211	MMF	Present	DPT Hepatitis B	15	30.1/able to walk, but muscle weakness
7	40.6/F	Localized swelling & palpable subcutaneous mass		R/O Vascular malformation	ND	Subcutaneous tissue over quadriceps: Pseudolymphoma	Present	DPT Hepatitis B	34	42.0/no muscle weakness

*GA* gestational age, *ND* not done.

*POMGNT1 c.T1702C:p.W568R/c.945dupT:p.D316_G317delinsX was found on whole exome sequencing.

**FKTN: no mutation of fukutin gene on congenital myopathy gene panel study.

The biopsy site for six patients was the quadriceps muscle, and in the remaining one patient, it was the subcutaneous tissue overlying the quadriceps since sonography revealed no lesions in the muscle. Six patients had various suspected clinical impressions, including mitochondrial disorders (cases 1, 3, and 4), Fukuyama congenital muscular dystrophy (case 2), congenital myopathy (case 5), autoimmune encephalitis (case 4), and spinal muscular atrophy, or congenital muscular dystrophy (case 6). All patients were vaccinated against HBV, HAV, and TT 4–12 months before the muscle biopsy. Table 2 contains detailed vaccination history.

**Table 2:** Summary of the vaccination history.

Case	Gestation age (weeks)	Birth weight (g)	Biopsy date	Birth date	HVA-date	HAV duration to the biopsy (months)	HBV-date	HBV duration to the biopsy (months)	DTP-date	DTP duration to the biopsy (months)	Biopsy site/vaccination site
1	30 + 5	1,470	2015-07	2014-05	2015–05	2	2014-11	8	2014-11	8	Thigh/thigh
2	38 + 0	3,620	2016-02	2015-05	ND	ND	2015-11	3	2015-11	4	Thigh/thigh
3	38 + 6	2,880	2016-09	2015-10	ND	ND	2016-04	5	2016-04	5	Thigh/thigh
4	38 + 0	2,900	2017-06	2015-01	2016-05	13	2015-07	23	2016-05	13	Thigh/thigh
5	38 + 4	3,030	2018-09	2017-06	2018-06	3	2017-12	9	2017-12	9	Thigh/thigh
6	38 + 1	3,005	2018-12	2017-03	2018-06	6	2017-09	15	2018-06	6	Thigh/thigh
7	Unknown	Unknown	2019-07	2016-03	2017-07	24	2016-09	34	2017-07	24	Thigh/thigh
Average		2,818				9.6 (3–13)		13.9 (3–34)		9.9 (4–24)	

*ND* not done.

**Figure 1:**
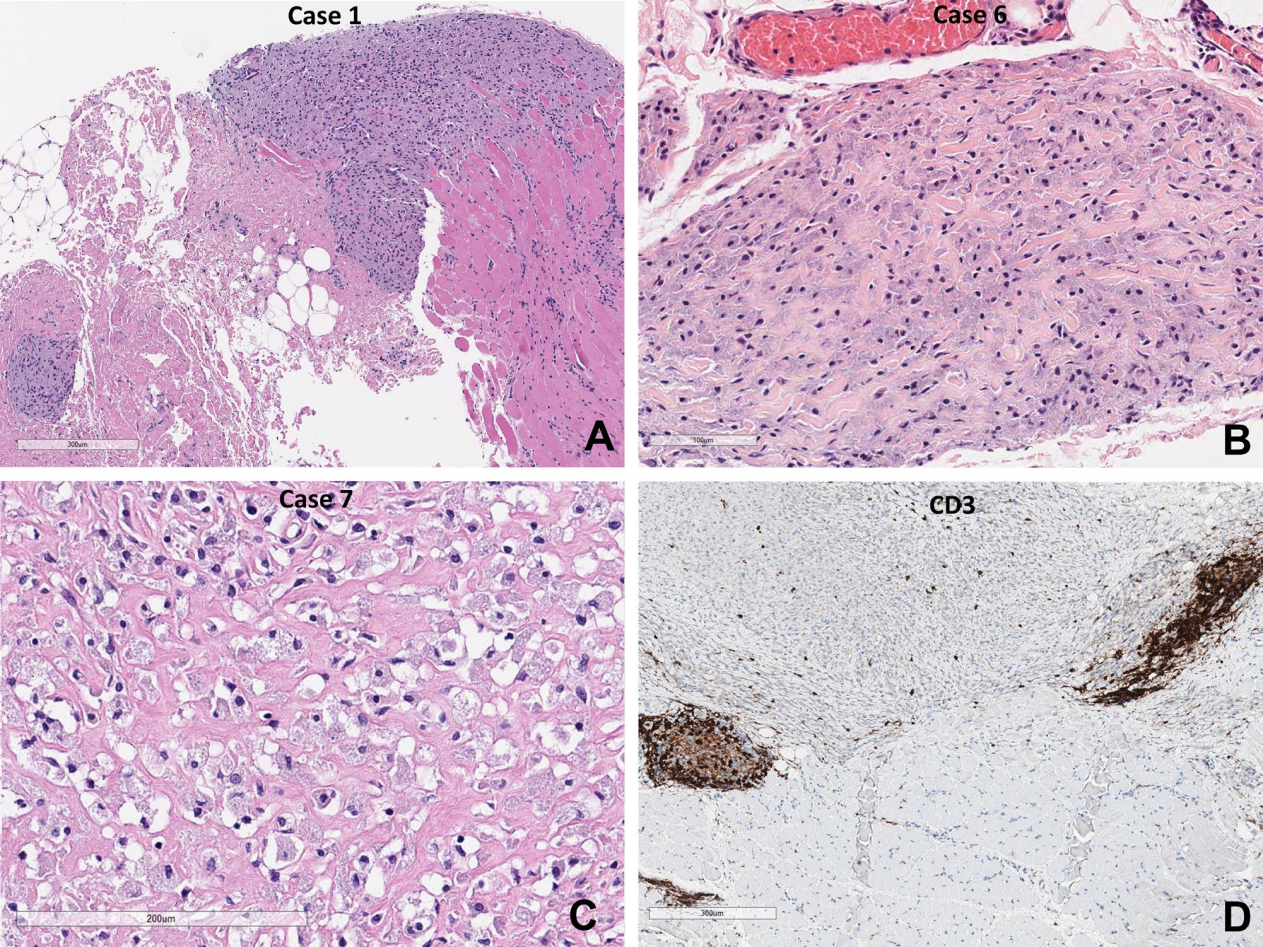
(**A**, **B**) Quadriceps muscle biopsies of the patients (case 1 and 6) show profound macrophage infiltration in the perimysium and epimysium. (**C**) High power view of infiltrating macrophages of the case 7 shows basophilic granular cytoplasm with pericellular lace-like fibrosclerosis. (**D**) CD3 immunohistochemistry reveals perivascular T-lymphocytic infiltration and a few scattered T-lymphocytes in the aggregate of the macrophages (**A**–**C**: H&E, **D**: CD3 immunohistochemistry, Scale bar: **A**, **D**: 300 μm, **B**: 100 μm, **C**: 200 μm).

**Figure 2:**
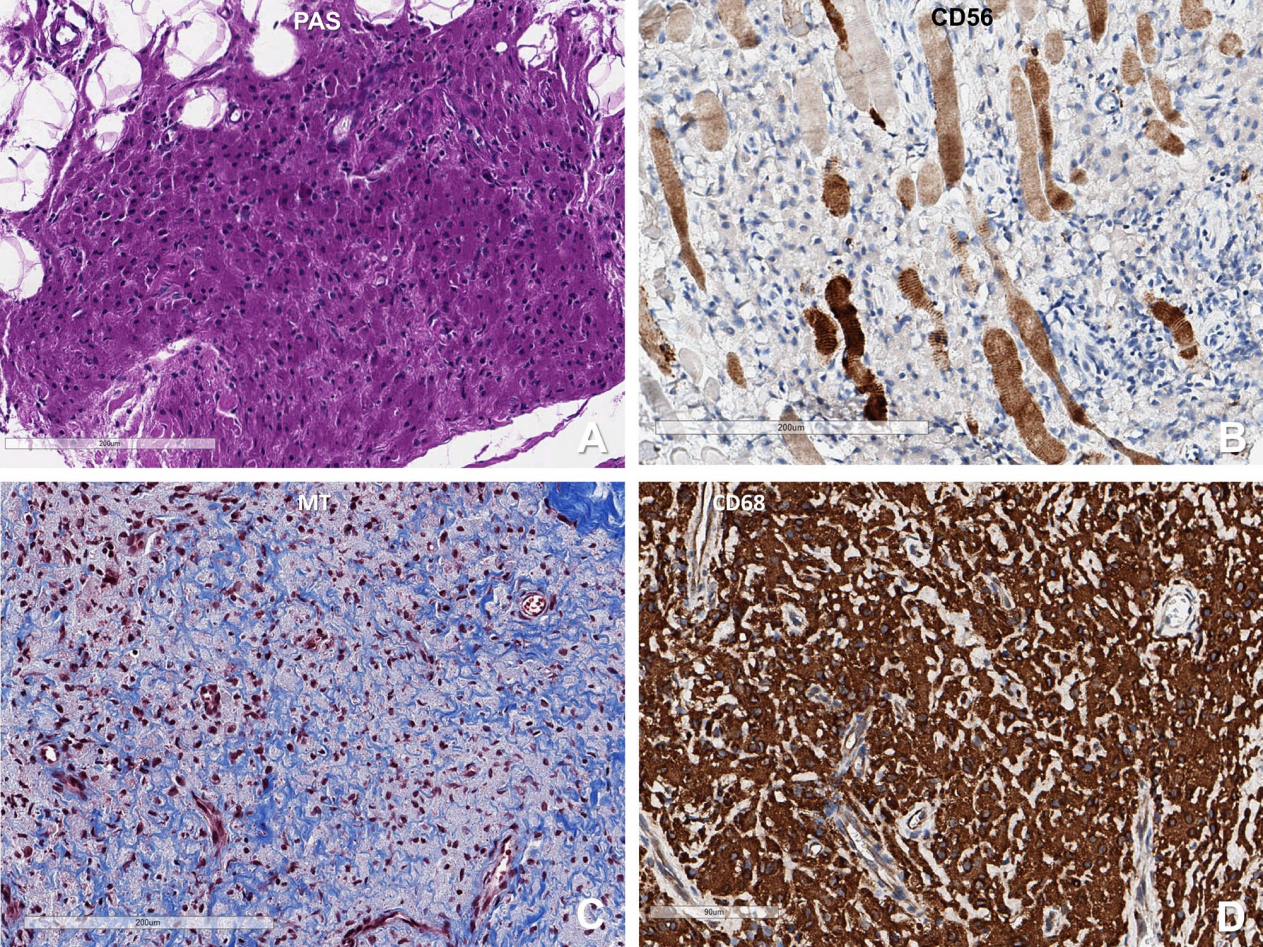
(**A**) PAS stain shows the purple colour of the macrophages. (**B**) The muscle fibres located adjacent to the macrophage infiltration area degenerate and atrophic, which are positive for CD56. (**C**) Masson trichrome stain reveals the blue collagenous stroma surrounding individual macrophages. (**D**) These macrophages are robustly positive for CD68 (**A**, **B**: H&E, **C**: PAS, **D**: CD68 immunohistochemistry). G) Macrophages are positive for CD68. (**A**: H&E, **B**: CD56, **C**: CD68, **D**: CD3) (Scale bar: **A**–**C**: 200 µm, **D**: 90 µm).

**Figure 3:**
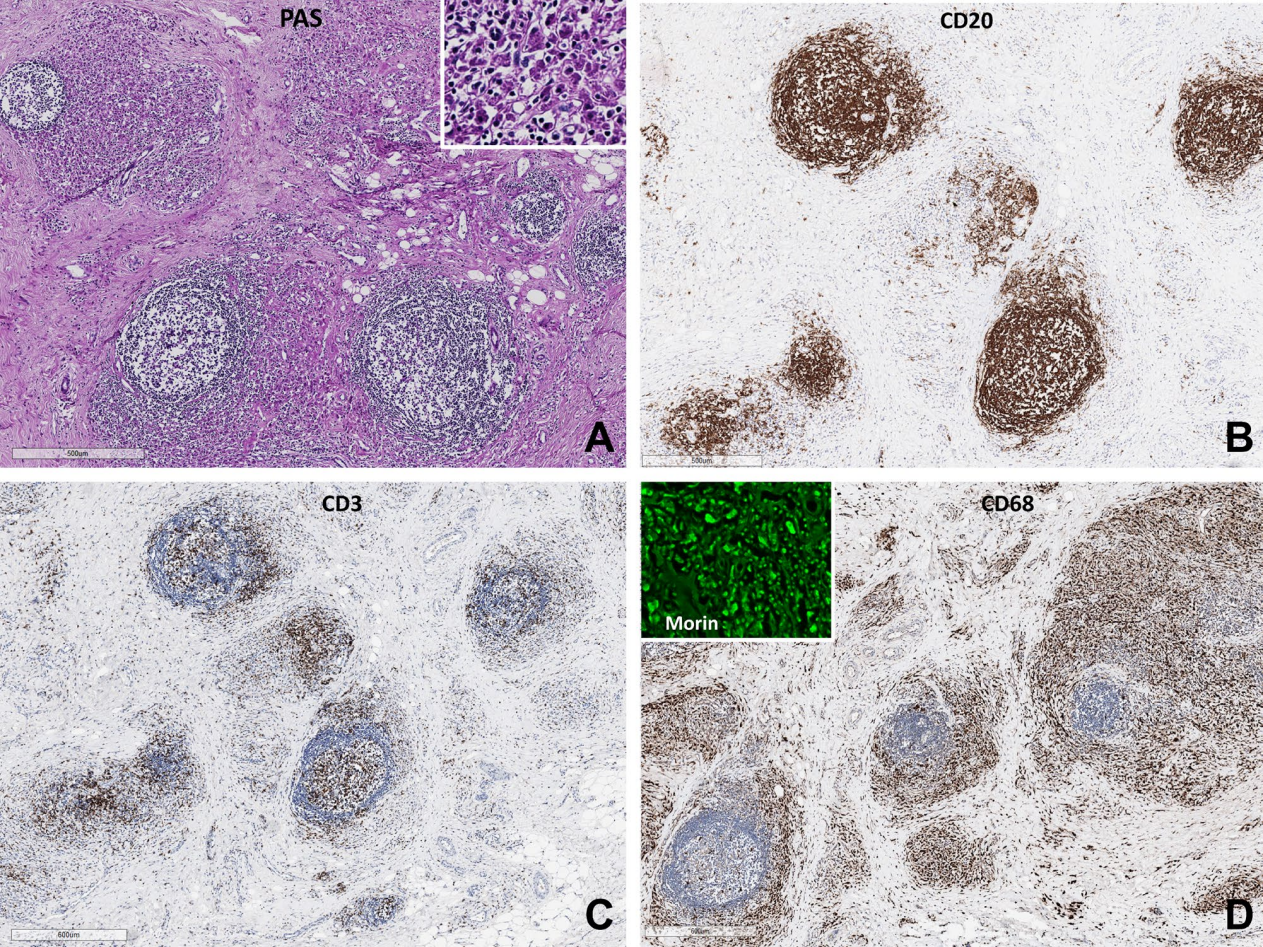
The pseudolymphomatous pathology of case 7 is shown in (**A**–**D**). (**A**) The follicle formation and infiltration of the perifollicular aluminium containing macrophages are seen in PAS stain. The inlet is the high power view of the PAS-positive, granular aluminium-containing macrophages. (**B**) The lymphoid follicles are delineated with CD20 immunostain. (**C**) The massive infiltration of macrophage infiltration remarkably shrinks the parafollicular T-zone. (**D**) The rings of typical aluminium-loaded macrophages around the tertiary lymphoid follicles are prominent with CD68 immunostain. Inlet is Morin stained section which shows strong green fluorescent cytoplasmic aluminium (Scale bar **A**, **B**: 500 μm, **C**, **D**: 600 μm, an inlet in A and D: X400).

The lesions contained a densely packed sheet of large polygonal-shaped macrophages, mainly in the perifascicular (perimysial) area, but also in the epimysium, and endomysium. Degeneration of the muscle fibers closest to the macrophage infiltration region was observed. (Fig. 1). The macrophages had a large granular cytoplasm, which appeared basophilic in H&E staining, and the granules were positive for PAS in all cases (Fig. 2). Individual macrophages were surrounded by a collagenous stroma, which was well delineated by Masson trichrome staining (Fig. 2). Infiltrating macrophages were positive for CD68, and the degenerated myofibers were positive for CD56 (Fig. 2). Perivascular lymphocytic aggregation was also present, and these lymphocytes were mostly CD3-positive T-cells, although CD4-, CD8-, and CD20-positive lymphocytes were also present. In the case of pseudolymphoma, the follicular organization was elegantly evidenced with CD20 immnostain. The rings of characteristic aluminium-loaded macrophages around the tertiary lymphoid follicles were prominent with CD68 immunostaining (Fig. 3). The Morin stain revealed strong green fluorescent cytoplasmic aluminium (Fig. 3).

**Figure 4:**
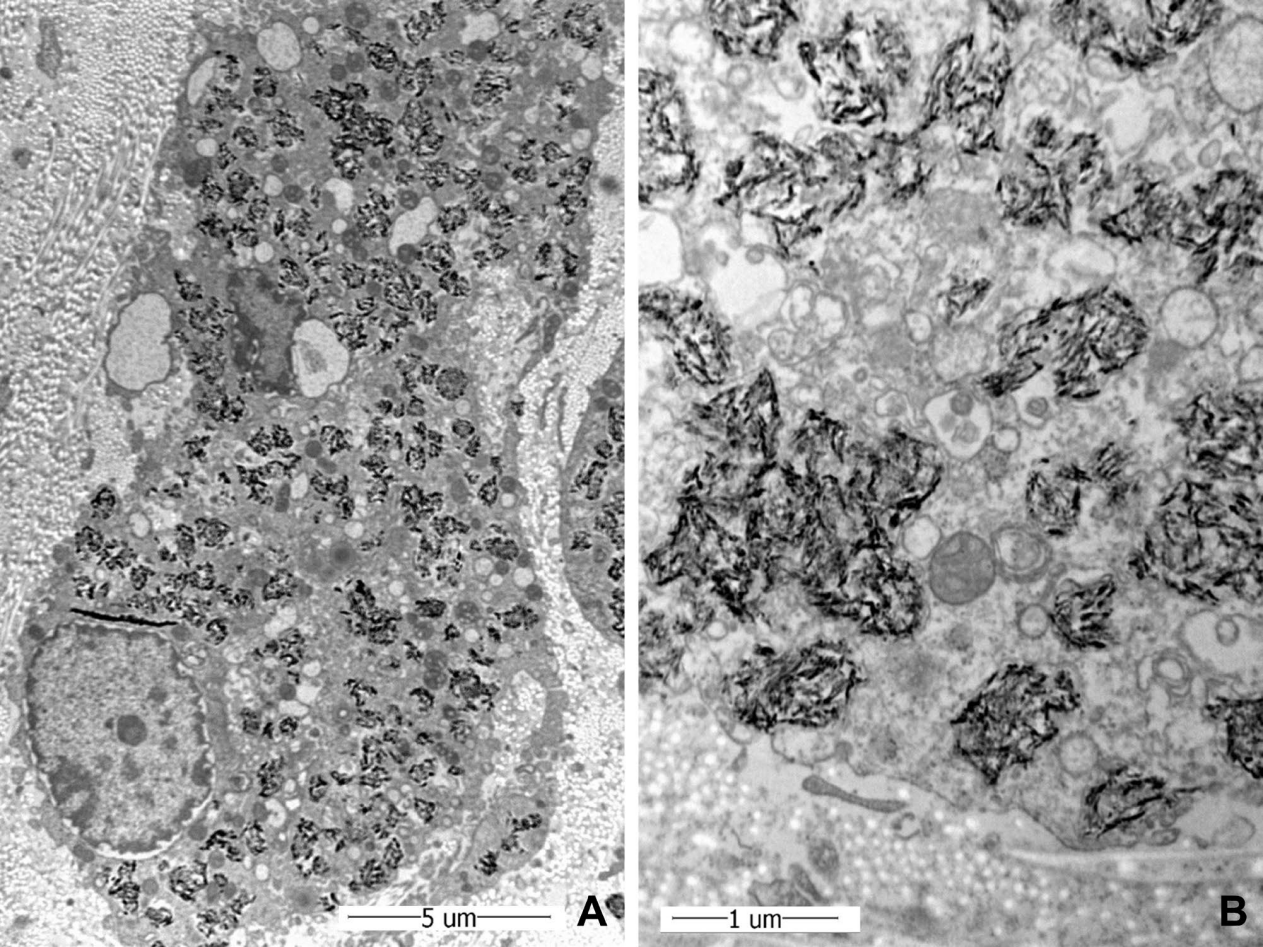
(**A**) Ultrastructurally, infiltrating macrophages have collagenous stroma. The cytoplasm of macrophages contains clusters of numerous crystalline inclusions, which may or may not be surrounded by lysosomal membranes. (**B**) These inclusions show electron-dense spiculated appearance consistent with aluminium hydroxide, which is the most common adjuvant in the vaccines [Uranyl acetate and lead citrate, (**A**: × 6,000, **B**: 25,000). [Scale bar: (**A**: 5 µm, **B**: 1 µm)].

The electron microscopy findings were unique and specific, which revealed the infiltration of numerous large macrophages into the perimysium or endomysium of the muscle. The cytoplasm of the macrophages contained clusters of spiculated electron-dense inclusions, consistent with aluminium hydroxide particles (Fig. 4)^4,6,9,17^. Some particles were enveloped by lysosomal membranes, whereas others appeared without a membrane. The muscle fibres had a degenerated and/or atrophic appearance, indicating loss or disorganisation of myofilaments. These findings were consistent with MMF.

## Discussion

Aluminium adjuvants are established in the vaccine repository owing to their pro-phagocytic and pro-inflammatory activation properties, which result in the stimulation of innate and acquired immune responses and complementary systems^1^. However, adjuvants based on crystalline aluminium hydroxide contain aluminium-coated particles of low biodegradability, which can cause side effects. After Gherardi et al. first reported 14 French adult patients (median age: 43 years, range 30–70 years old, male: female = 6:8) with MMF in 1988, various adverse effects of aluminium hydroxide-containing vaccines have been reported (Supplementary Table 2)^6,18^. Several childhood cases have also been reported (median age: 20 months, range 4–70 months, male:female = 2:1)^2,4,5,7,8^.

The most common organic lesion associated with aluminium-based vaccination is MMF. The term ‘MMF’ designates the histopathological lesion it-self, but clinically, it is characterised by myalgia, arthralgia, muscle weakness, or profound asthenia, and fever^1,3,6,19^. Furthermore, myalgic encephalomyelitis, cognitive dysfunction, and aluminium neurotoxicity or neuropsychological symptoms, such as demyelinating CNS disorder, have been reported^20–23^. Hypotonia, delayed motor milestones, seizures, and irritability are common symptoms in children^5,7,8^. Autoimmune (autoinflammatory) syndrome induced by adjuvants (ASIA)^12,13,24^ and Gulf War syndrome (GWS)-chronic fatigue syndrome, which can develop in soldiers subjected to multiple aluminium-containing vaccinations, are known to be associated with post-vaccinal disseminated encephalomyelitis^14,15^. Specific histological findings and epidemiological studies suggest that even Alzheimer's disease and autism spectrum disorder may be associated with aluminium-based vaccination, although these assumptions are currently debatable^2^. Couette et al. reported the unexpected death during sleep of a 37-years-old MMF patient, but the cause of death was not defined because an autopsy was rejected^22^. Blanc-Durand et al. verified the statistical significance of the uniquely localised cerebral hypometabolism in the temporo-occipital cortex and cerebellum of patients with MMF compared to healthy individuals, by performing computer-aided support vector machine (SVM) classification on large and multicentric^18^F-FDG-PET cohorts^25^.

Therefore, MMF is not a simple local disease, but rather a systemic, complicated, and severe disease. The exact pathology of the systemic manifestation of MMF should be investigated through autopsy. However, no autopsy-proven MMF has been reported so far.

MMF lesions in our patients were found in vaccination sites, i.e., the quadriceps; however, these patients presented with a severe systemic illness, such as a delayed motor milestone or muscle weakness, congenital hypotonia, etc. (n = 1) (Table 1), which is consistent with the reported clinical manifestations of MMF patients^2,5,7,8^. However, sensorineural hearing loss (n = 1) and tracheo-broncho-laryngomalacia (n = 1) may result from superimposed coincidental diseases and may not be associated with MMF. During the follow-up period, patients showed disability, and 67% (4/6) were unable to walk. In one patient, the lesion was localized to the subcutaneous tissue overlying the quadriceps, and the patient did not have muscle weakness or systemic illness. According to previous reports, the quadriceps muscle is the most frequent site affected by MMF after the deltoid muscle^3,26^. Typical histopathology with ultrastructural features of spiculated inclusions in the macrophages suggested an association with aluminium hydroxide, which is the most commonly used adjuvant in vaccines.

The mechanism of MMF and the resulting systemic illness may be linked to the high biopersistence of aluminium hydroxide in the body. Dendritic cells and macrophages promptly phagocytise these aluminium particles, which enter the draining lymph nodes after muscle injection^3^. Then, as the phagocytic cells circulate, the particles can disseminate throughout the body and accumulate in various organs, including the brain^16,27^. The exact definition of long-term persistence is not yet clear. In adult cases, symptoms have been delayed from several months to years after vaccination, but some of the reported children began the symptoms immediately after vaccination and lasted for a long time.^5^

The most remarkable pathologies in our cases were: 1) massive infiltration of basophilic, granular and PAS-positive, large macrophages leading to fibrosclerosis in the epimysium, perimysium, endomysium, and subcutaneous tissue in the injection site, and 2) secondary muscle degeneration at the macrophage infiltrated area, as previously reported.^6,28,29^ These characteristics indicate aluminium-phagocytic macrophage infiltration and muscle damage by these macrophages in the injection site.

Ultrastructural intracytoplasmic spiculated inclusions of macrophages with collagen deposits form a pathognomonic feature of MMF. The ultrastructural confirmation is the best method for diagnosis in addition to Morin staining for aluminium^30^. In a previously established animal model of MMF, these muscle pathologies associated with chronic fatigue syndrome (CFS) were found by intramuscular injection of an aluminium hydroxide-based adjuvant^31^.

MMF patients often visit the paediatric neurology clinic, but the condition may be clinically under-recognised. Indeed, the clinical differential diagnoses of our seven MMF patients did not include MMF because of the presence of other severe clinical manifestations, such as severe delay of motor milestones, muscle weakness, inability to walk, and other accompanying central nervous system (CNS) lesion-associated symptoms (e.g., dysarthria, instability, or mental retardation). In particular, delayed motor milestones have been described in infants and young children with MMF^4,8^. Previous reports indicated that clinical manifestations presented in MMF patients might have been due to the dissemination and accumulation of aluminium-containing phagocytic macrophages and consequent inflammation of the CNS, as mentioned above^3,4,32^. Animal studies have shown that aluminium-adjuvanted vaccines have induced MMF-like massive macrophagic infiltration and myofiber degeneration in the injection site of the muscle.^33^ The macrophagic lesions have shrunk with time, but the lesion size was considerably depending on the genetic background^33^. However, this animal study did not evaluate tissues or organs that were not injected. It is uncertain that these clinical symptoms can occur purely circumstantially, regardless of MMF, so further research is needed to determine the direct relationship between these clinical symptoms and MMF.

In conclusion, we described six cases of MMF and one case of subcutaneous pseudolymphoma. Paediatricians and pathologists should recognise this uncommon condition to avoid misdiagnosis and mistreatment, and pharmaceutical companies should develop better adjuvants, based on compounds other than aluminium, which do not cause such adverse effects. As for the susceptibility and verification of the neurotoxicity of aluminium-based vaccination, including the development of Alzheimer’s disease and autism spectrum disorder, future well-designed studies are required.

## Supplementary information

Supplementary information.
