# Supplementary material for: Macrophagic myofasciitis and subcutaneous pseudolymphoma caused by aluminium adjuvants
Source: Sci Rep. 2020 Jul 16;10:11834. doi: 10.1038/s41598-020-68849-8 (PMC7366910; doi:10.1038/s41598-020-68849-8)
Supplement: Supplementary file 1 — Supplementary information. [file 41598_2020_68849_MOESM1_ESM.docx]

**Macrophagic myofasciitis and subcutaneous pseudolymphoma caused by Aluminium adjuvant**

Hyunhee Kim^1^, M.D., Ka Young Lim^1^ M.D., Jeongwan Kang^1^, M.D. Jin Woo Park^1^, M.D., and Sung-Hye Park^1,2,3^ M.D., Ph.D

^1^Department of Pathology, Seoul National University Hospital, Seoul 03080, Republic of Korea

^2^Department of Pathology, Seoul National University College of Medicine, Seoul 03080, Republic of Korea

^2^Institute of Neuroscience, Seoul National University College of Medicine, Seoul 03080, Republic of Korea

Supplementary Table 1. The information of the primary antibodies used in this study.

| Antibody | Dilution | Company |
| --- | --- | --- |
| CD68 | 1: 500 | DAKO, Glostrup, Denmark |
| CD3 | 1: 300 | Roche (Ventana), Export, PA, USA |
| CD20 | 1: 500 | DAKO, Glostrup, Denmark |
| CD4 | Ready to use | Roche (Ventana), Export, PA, USA |
| CD8 | Ready to use | Novocastra, Newcastle, UK |
| CD56 | 1: 100 | Life Technology, Carlsbad, CA, USA |

Supplementary Table 2. Previously reported key papers about aluminium-hydroxide based vaccine induced diseases or symptoms

| Disease or symptoms associated aluminium hydroxide adjuvant of vaccines | Representative references |
| --- | --- |
| Macrophage myofasciitis (MMF) | ^1-6^ |
| Chronic fatique syndrome | ^1,7-11^ |
| Neurotoxicity | ^1,12-15^ |
| Association with Alzheimer’s disease (AD) or autism spectrum disease (ASD) | ^3,16-18^ |
| Autoimmune/inflammatory syndrome induced by adjuvant (ASIA) | ^7,19-25^ |
| Gulf war syndrome | ^26-28^ |
| Systemic dissemination of aluminium containing macrophages | ^13,29,30^ |
| Myalgia and motor weakness | ^1,2,14,31^ |
| Hypotonia | ^29,32-35^ |
| Delayed motor milestones | ^17,29,32,34,36^ |
| Difficulties in sitting, standing or walking | ^2,34,37,38^ |
| Ultrastructure of MMF | ^5,29,38-42^ |
| Morin stain of aluminium hydroxide (immunofluorescence stain) | ^2,39,43,44^ |
| Criteria of MMF | ^14,31^ |

References

1. Gherardi, R.K., Crepeaux, G. & Authier, F.J. Myalgia and chronic fatigue syndrome following immunization: macrophagic myofasciitis and animal studies support linkage to aluminum adjuvant persistency and diffusion in the immune system. *Autoimmun Rev* **18**, 691-705 (2019).

2. Soares Santos, D., Santos, A., Rebelo, O. & Santos, R.M. Macrophagic myofasciitis: a challenging diagnosis. *BMJ Case Rep* **2018**(2018).

3. Principi, N. & Esposito, S. Aluminum in vaccines: Does it create a safety problem? *Vaccine* **36**, 5825-5831 (2018).

4. He, P., Zou, Y. & Hu, Z. Advances in aluminum hydroxide-based adjuvant research and its mechanism. *Hum Vaccin Immunother* **11**, 477-488 (2015).

5. Gherardi, R.K.*, et al.* Macrophagic myofasciitis: an emerging entity. Groupe d'Etudes et Recherche sur les Maladies Musculaires Acquises et Dysimmunitaires (GERMMAD) de l'Association Francaise contre les Myopathies (AFM). *Lancet* **352**, 347-352 (1998).

6. Cherin, P. & Gherardi, R.K. Emergence of a new entity, the macrophagic myofasciitis. GERMMAD Study Group of the French Association Against Myopathies. Study and Research Group on Acquired Dysimmunity-related Muscle Disease. *Rev Rhum Engl Ed* **65**, 541-542 (1998).

7. Polido Pereira, J., Barroso, C., Evangelista, T., Fonseca, J.E. & Pereira da Silva, J.A. Macrophagic myofasciitis: a case report of autoimmune/inflammatory syndrome induced by adjuvants (ASIA). *Acta Reumatol Port* **36**, 75-76 (2011).

8. Authier, F.J.*, et al.* Chronic fatigue syndrome in patients with macrophagic myofasciitis. *Arthritis Rheum* **48**, 569-570 (2003).

9. Loyo, E., Jara, L.J., Lopez, P.D. & Puig, A.C. Autoimmunity in connection with a metal implant: a case of autoimmune/autoinflammatory syndrome induced by adjuvants. *Auto Immun Highlights* **4**, 33-38 (2013).

10. Nancy, A.L. & Shoenfeld, Y. Chronic fatigue syndrome with autoantibodies--the result of an augmented adjuvant effect of hepatitis-B vaccine and silicone implant. *Autoimmun Rev* **8**, 52-55 (2008).

11. Appel, S., Chapman, J. & Shoenfeld, Y. Infection and vaccination in chronic fatigue syndrome: myth or reality? *Autoimmunity* **40**, 48-53 (2007).

12. Piyasirisilp, S. & Hemachudha, T. Neurological adverse events associated with vaccination. *Curr Opin Neurol* **15**, 333-338 (2002).

13. Gherardi, R.K., Eidi, H., Crepeaux, G., Authier, F.J. & Cadusseau, J. Biopersistence and brain translocation of aluminum adjuvants of vaccines. *Front Neurol* **6**, 4 (2015).

14. Aoun Sebaiti, M.*, et al.* Cognitive dysfunction associated with aluminum hydroxide-induced macrophagic myofasciitis: A reappraisal of neuropsychological profile. *J Inorg Biochem* **181**, 132-138 (2018).

15. Passeri, E.*, et al.* Long-term follow-up of cognitive dysfunction in patients with aluminum hydroxide-induced macrophagic myofasciitis (MMF). *J Inorg Biochem* **105**, 1457-1463 (2011).

16. Geier, D.A., King, P.G. & Geier, M.R. Mitochondrial dysfunction, impaired oxidative-reduction activity, degeneration, and death in human neuronal and fetal cells induced by low-level exposure to thimerosal and other metal compounds. *Toxicol Environ Chem* **91**, 735-749 (2009).

17. Tomljenovic, L. & Shaw, C.A. Do aluminum vaccine adjuvants contribute to the rising prevalence of autism? *J Inorg Biochem* **105**, 1489-1499 (2011).

18. Shaw, C.A., Li, Y. & Tomljenovic, L. Administration of aluminium to neonatal mice in vaccine-relevant amounts is associated with adverse long term neurological outcomes. *J Inorg Biochem* **128**, 237-244 (2013).

19. Vincent, D.*, et al.* Association of macrophagic myofasciitis and fibromuscular dysplasia with renal fibromuscular dysplasia: first case report. *Clin Exp Rheumatol* **18**, 753-754 (2000).

20. Cadusseau, J.*, et al.* Selective elevation of circulating CCL2/MCP1 levels in patients with longstanding post-vaccinal macrophagic myofasciitis and ASIA. *Curr Med Chem* **21**, 511-517 (2014).

21. Cruz-Tapias, P., Agmon-Levin, N., Israeli, E., Anaya, J.M. & Shoenfeld, Y. Autoimmune (auto-inflammatory) syndrome induced by adjuvants (ASIA)--animal models as a proof of concept. *Curr Med Chem* **20**, 4030-4036 (2013).

22. Esposito, S.*, et al.* Autoimmune/inflammatory syndrome induced by adjuvants (ASIA): clues and pitfalls in the pediatric background. *Immunol Res* **60**, 366-375 (2014).

23. Perricone, C.*, et al.* Autoimmune/inflammatory syndrome induced by adjuvants (ASIA) 2013: Unveiling the pathogenic, clinical and diagnostic aspects. *J Autoimmun* **47**, 1-16 (2013).

24. Shoenfeld, Y. & Agmon-Levin, N. 'ASIA' - autoimmune/inflammatory syndrome induced by adjuvants. *J Autoimmun* **36**, 4-8 (2011).

25. Jara, L.J., Medina, G., Gomez-Banuelos, E., Saavedra, M.A. & Vera-Lastra, O. Still's disease, lupus-like syndrome, and silicone breast implants. A case of 'ASIA' (Shoenfeld's syndrome). *Lupus* **21**, 140-145 (2012).

26. Hotopf, M.*, et al.* Role of vaccinations as risk factors for ill health in veterans of the Gulf war: cross sectional study. *BMJ* **320**, 1363-1367 (2000).

27. Theeler, B.J., Simper, N.B. & Ney, J.P. Polyglandular autoimmunity with macrophagic myofasciitis. *Clin Rheumatol* **27**, 667-669 (2008).

28. Hotopf, M.*, et al.* Risk factors for continued illness among Gulf War veterans: a cohort study. *Psychol Med* **34**, 747-754 (2004).

29. Nevo, Y.*, et al.* Childhood macrophagic myofasciitis-consanguinity and clinicopathological features. *Neuromuscul Disord* **14**, 246-252 (2004).

30. Preusse, C.*, et al.* Th2-M2 immunity in lesions of muscular sarcoidosis and macrophagic myofasciitis. *Neuropathol Appl Neurobiol* **41**, 952-963 (2015).

31. Alijotas-Reig, J. Human adjuvant-related syndrome or autoimmune/inflammatory syndrome induced by adjuvants. Where have we come from? Where are we going? A proposal for new diagnostic criteria. *Lupus* **24**, 1012-1018 (2015).

32. Gruis, K.L., Teener, J.W. & Blaivas, M. Pediatric macrophagic myofasciitis associated with motor delay. *Clin Neuropathol* **25**, 172-179 (2006).

33. Israeli, E., Agmon-Levin, N., Blank, M. & Shoenfeld, Y. Macrophagic myofaciitis a vaccine (alum) autoimmune-related disease. *Clin Rev Allergy Immunol* **41**, 163-168 (2011).

34. Kakkar, A.*, et al.* Childhood macrophagic myofasciitis: A series from the Indian subcontinent. *Muscle Nerve* **56**, 71-77 (2017).

35. Rivas, E.*, et al.* Macrophagic myofasciitis in childhood: a controversial entity. *Pediatr Neurol* **33**, 350-356 (2005).

36. Lach, B. & Cupler, E.J. Macrophagic myofasciitis in children is a localized reaction to vaccination. *J Child Neurol* **23**, 614-619 (2008).

37. Bornemann, A.*, et al.* July 2003: 62-year-old female with progressive muscular weakness. *Brain Pathol* **14**, 109-110, 115 (2004).

38. Kalil, R.K.*, et al.* Macrophagic myofasciitis in childhood: the role of scanning electron microscopy/energy-dispersive spectroscopy for diagnosis. *Ultrastruct Pathol* **31**, 45-50 (2007).

39. Chkheidze, R.*, et al.* Morin Stain Detects Aluminum-Containing Macrophages in Macrophagic Myofasciitis and Vaccination Granuloma With High Sensitivity and Specificity. *J Neuropathol Exp Neurol* **76**, 323-331 (2017).

40. Shivane, A., Hilton, D.A., Moate, R.M., Bond, P.R. & Endean, A. Macrophagic myofasciitis: a report of second case from UK. *Neuropathol Appl Neurobiol* **38**, 734-736 (2012).

41. Shingde, M., Hughes, J., Boadle, R., Wills, E.J. & Pamphlett, R. Macrophagic myofasciitis associated with vaccine-derived aluminium. *Med J Aust* **183**, 145-146 (2005).

42. Gherardi, R.K.*, et al.* Macrophagic myofasciitis lesions assess long-term persistence of vaccine-derived aluminium hydroxide in muscle. *Brain* **124**, 1821-1831 (2001).

43. Eidi, H.*, et al.* Fluorescent nanodiamonds as a relevant tag for the assessment of alum adjuvant particle biodisposition. *BMC Med* **13**, 144 (2015).

44. Guillard, O.*, et al.* Aluminium overload after 5 years in skin biopsy following post-vaccination with subcutaneous pseudolymphoma. *J Trace Elem Med Biol* **26**, 291-293 (2012).
